# Altered Islet Morphology but Normal Islet Secretory Function *In Vitro* in a Mouse Model with Microvascular Alterations in the Pancreas

**DOI:** 10.1371/journal.pone.0071277

**Published:** 2013-07-29

**Authors:** Elena Kostromina, Xiaorui Wang, Weiping Han

**Affiliations:** 1 Singapore Bioimaging Consortium, Agency for Science, Technology and Research, Singapore, Singapore; 2 Institute of Molecular and Cell Biology, Agency for Science, Technology and Research, Singapore, Singapore; 3 Department of Biochemistry, Yong Loo Lin School of Medicine, National University of Singapore, Singapore, Singapore; 4 Cardiovascular and Metabolic Disorders Program, Duke-NUS Graduate Medical School, Singapore, Singapore; University of Hong Kong, China

## Abstract

**Background:**

Our previous studies have shown that signal transducer and activator of transcription 3 (STAT3) signaling is important for the development of pancreatic microvasculature via its regulation of vascular endothelial growth factor-A (VEGF-A). Pancreas-specific STAT3-KO mice exhibit glucose intolerance and impaired insulin secretion *in vivo*, along with microvascular alterations in the pancreas. However, the specific role of STAT3 signaling in the regulation of pancreatic islet development and function is not entirely understood.

**Methodology/Principal Findings:**

To investigate the role of STAT3 signaling in the formation and maintenance of pancreatic islets, we studied pancreas-specific STAT3-KO mice. Histological analysis showed that STAT3 deficiency affected pancreatic islet morphology. We found an increased proportion of small-sized islets and a reduced fraction of medium-sized islets, indicating abnormal islet development in STAT3-KO mice. Interestingly, the islet area relative to the whole pancreas area in transgenic and control mice was not significantly different. Immunohistochemical analysis on pancreatic cryosections revealed abnormalities in islet architecture in STAT3-KO mice: the pattern of peripheral distribution of glucagon-positive α-cells was altered. At the same time, islets belonging to different size categories isolated from STAT3-KO mice exhibited normal glucose-stimulated insulin secretion in perifusion experiments *in vitro* when compared to control mice.

**Conclusions:**

Our data demonstrate that STAT3 signaling in the pancreas is required for normal islet formation and/or maintenance. Altered islet size distribution in the KO mice does not result in an impaired islet secretory function *in vitro*. Therefore, our current study supports that the glucose intolerance and *in vivo* insulin secretion defect in pancreas-specific STAT3-KO mice is due to altered microvasculature in the pancreas, and not intrinsic beta-cell function.

## Introduction

Insulin secretion and its regulation by glucose and other physiological stimuli are vital to the maintenance of glucose homeostasis. The highly regulated insulin secretion process is determined by both the secretory function of β-cells and the microcirculation in the endocrine pancreas [1], [2], [3]. The unique structural organization of pancreatic islets, composed of endocrine cells and a highly tortuous and dense islet microvascular network, ensures efficient glucose sensing, adequate insulin release into the circulatory system and timely delivery of insulin to target tissues [4], [5], [6]. The development and maintenance of pancreatic islets is a complex process, which requires multiple transcription factors that are involved in regulation of the expression of key genes during pancreas development [7], [8]. However, the role of all these factors in islet formation, growth, and function is not entirely understood.

Signal transducer and activator of transcription 3 (STAT3) is a significant signaling protein essential for many cellular events including cell growth and apoptosis. STAT3 is activated in response to various growth factors and cytokines and mediates the expression of a number of genes in response to the stimuli [9], [10], [11], [12]. For example, STAT3 signaling plays a direct role in the regulation of vascular endothelial growth factor-A (VEGF-A) expression [13], and its activation is essential for VEGF-A overexpression in pancreatic cancer, a key regulator in abnormal tumor angiogenesis [14]. Using a mouse model with Pdx-Cre–mediated STAT3 inactivation [15] we showed that pancreatic STAT3 signaling is essential for the development of highly organized islet microvascular network through its regulation of VEGF-A expression under normal physiological conditions. Under the Pdx1 promoter, Cre expression in the pancreatic epithelium starts at early embryonic stages [16], which ensures STAT3 deletion prior to islet formation. In the absence of pancreatic STAT3-signaling from very early stages of development in pancreas-specific STAT3-knockout mice (p-KO), VEGF-A production is substantially reduced, and the density of the microvascular bed in islets is significantly decreased [15]. P-KO mice exhibit glucose intolerance and impaired glucose-stimulated insulin secretion *in vivo*; however, evaluation of islet function in perifusion experiments did not show significant β-cell secretory defects in response to glucose challenge *in vitro*. A dichotomy of impaired glucose-stimulated insulin secretion *in vivo* and normal islet secretory function *in vitro*, as revealed in our p-KO mice, has also been reported for mice lacking VEGF-A signaling in pancreatic β-cells [17]. High VEGF-A levels in islet endocrine cells are required for the normal development of islet capillaries composed of thin and fenestrated endothelial cells separated from pancreatic beta cells by a basement membrane [16]. Insulin is released from the secretory granules of the pancreatic beta cells into the circulatory system through the basement membrane and endothelial fenestrae [18]. VEGF-A deficient pancreatic islets had a decreased number of capillaries and substantially altered morphology in the remaining capillaries including a decreased number of fenestrae and many caveolae in the endothelial cells, however, secretory granules and plasma membranes in the beta cells were not altered [16]. Abnormal development of microvascular network in pancreatic islets, due to reduced expression of VEGF-A, is an important factor that affects insulin output from islets in response to secretagogues, leading to impaired insulin release *in vivo* without impairment of β-cell secretory function [19], [20].

Along with microvascular abnormalities in islets and glucose intolerance, disruption of pancreatic VEGF-A signaling is accompanied by alterations in islet formation in VEGF-A–KO mice [16]. A larger number of small islets and unchanged islet cell area are found in VEGF-A–KO mice. On the other hand, VEGF-A overexpression, under the control of Pdx-1 promoter, leads to hypervascularization of the pancreas as well as hyperplasia of pancreatic islets in transgenic mice [21]. Development of the endocrine pancreas is determined by signals from the blood vessel endothelium at an early stage of endocrine cell differentiation [21], and islets form next to blood vessels. The later stage of pancreas development includes paracrine VEGF-A signaling, which allows the formation of a branching capillary network in islets and interaction of islets with the circulatory system [16]. Lack of or excess blood vessels in islets do not substantially impair later-stage endocrine pancreas development but do have moderate effects on further development of endocrine cells [22]. These findings suggest that disruption of STAT3 and VEGF-A signaling pathways in the pancreas, along with microvascular alterations, may affect islet formation and, thus, insulin secretory function *in vivo*. However, whether a causal relationship exists between altered islet dimensions/architecture and impaired glucose tolerance has not been sufficiently studied, and further studies are needed to delineate the precise role of STAT3 in regulating islet development and function.

To address this question, we performed histological analysis of pancreata specimens from p-KO and control mice and evaluated the secretory function of isolated islets of different sizes *in vitro*. The results of morphological examination showed that p-KO mice exhibited abnormal islet size distribution, including an increased proportion of small islets and a decreased portion of medium-sized islets, though the islet area relative to the whole pancreas area was unchanged. The disruption of STAT3 signaling in the pancreas also led to an altered pattern of α-cell distribution within the islets of transgenic mice. Furthermore, islets with different sizes isolated from p-KO mice exhibited normal glucose-stimulated insulin secretion *in vitro* compared to control animals. Our findings suggest that STAT3 signaling in the pancreas is required for normal islet growth, development and architecture, but is not essential for normal islet secretory function *in vitro*.

## Materials and Methods

### Ethics statement

All animal experiments in this study were conducted in accordance with the guidelines for animal care and use established by the Institutional Animal Care and Use Committee (IACUC) of the Agency for Science, Technology and Research (A*STAR) in Singapore. The protocol was approved by the A*STAR IACUC (#050129 and #080351).

### Animal welfare

Only female mice were used in this study, and they were bred and housed in the animal facilities of the Biological Resource Centre under a 12 h light: 12 h darkness cycle with standard rodent diet (Harland Teklad Global Diets, USA) and water provided *ad libidum*.

### Pancreas-specific STAT3 KO mouse

Mice with pancreas-specific STAT3 deletion (p-KO) were generated by crossing STAT3^fl/fl^ mice containing *Cre* transgene under pdx1 promoter (Pdx1-Cre) with STAT3^fl/fl^ mice on the C57BL/6 genetic background as previously described [15]. STAT3^fl/+^ was a generous gift from Drs. Kiyoshi Takeda and Shizuo Akira (Osaka University) and Pdx1-Cre from Dr. Doug Melton (Harvard University). Genotyping was performed on genomic DNA extracted from tail biopsies by PCR analysis using the following set of primers: 5′-TGC TTC TGT CCG TTT GCC GGT-3′ and 5′-CTA AGT GCC TTC TCT ACA CCT-3′ for Pdx1-Cre (500 bp); 5′-CCT GAA GAC CAA GTT CAT CTG TGT GAC-3′ and 5′-CAC ACA AGC CAT CAA ACT CTG GTC TCC-3′ for STAT3 alleles (PCR fragment of 280 bp and 350 bp for wt and mutant allele, respectively).

### Immunohistochemistry and confocal microscopy

Pancreata harvested from p-KO and control mice were fixed in fresh 4% paraformaldehyde solution in PBS for 2–3 hours at 4°C, and after washing in PBS, incubated subsequently in 15% and 30% sucrose-PBS solution at 4°C during 24 hours [23]. After fixation the tissue specimens were embedded in Tissue-Tek optimum cutting temperature compound medium (Ted Pella, Redding, USA), frozen and stored at −80°C. For immunohistochemical staining 5-μm-thick cryosections mounted on glass slides (Polysine, Menzel GmbH, Germany) were washed in PBS with 0.1% Triton X-100, blocked with 5% goat serum for 30 min and incubated with primary antibodies overnight at 4°C. A polyclonal guinea pig anti-insulin antibody at 1∶200 dilution (Dako) and polyclonal rabbit anti-glucagon (Dako) at 1∶200 dilution were used for insulin and glucagon detection, respectively. After washing, a combination of secondary antibodies, Alexa Fluor 594 conjugated goat anti-rabbit Ig and Alexa Fluor 488 conjugated goat anti-guinea pig Ig was applied. Digital images of immunostained tissue sections were acquired using a Zeiss LSM510 META confocal laser scanning microscope (objectives 40×, 63×; Carl Zeiss, Germany).

Insulin content in pancreatic islets was assessed as the average pixel intensity of insulin positive staining per islet after background subtraction as previously described [24]. All images were acquired with identical settings from randomly selected pancreatic sections and analyzed using Image J software (NIH, USA).

### Histological analysis

For histological study, the dissected pancreata were fixed in 10% Neutral Buffered formalin solution at 4°C overnight and embedded in paraffin. 10 μm-thick sections stained with Haematoxylin and Eosin were examined under light microscope. Series of overlapping histological images were acquired from each histological section using a Motorized Research Nikon Microscope ECLIPSE 90i (objectives 20×, 4×). Full view of the entire pancreatic sections was achieved by computerized stitching of multiple single planes with the NIS Elements software (Nikon, Japan). Composite images of histological sections containing up to 80–90 overlapping single-plane images were further analyzed using Image Pro-Plus software (Media Cybernetics). To determine the ratio of islet area to the entire pancreatic area composite images obtained from 10 histological sections of the dorsal pancreas for each animal were used.

### Pancreatic islet isolation and insulin secretion

Pancreatic islets were isolated from p-KO and control mice by liberase digestion as previously described [25]. Isolated islets, hand-picked and sorted according to their size under stereomicroscope were cultured overnight in RPMI 1640 medium (Invitrogen) supplemented with 10% fetal calf serum, 2 mM L-glutamine, 15 mM HEPES, 1% streptomycin and penicillin and 11.1 mM glucose. Insulin release from pancreatic islets was measured in perifusion incubation with Krebs-Ringer bicarbonate (KRH) buffer, containing in mM: 130 NaCl, 4.7 KCl, 1.2 KH_2_PO_4_, 1.2 MgSO_4_, 2.56 CaCl_2_, 20 HEPES, 1 mg/ml BSA and supplemented with 3 mM glucose (basal) or 20 mM (stimulatory) glucose. After preliminary 1 hour incubation in KRH buffer, batches of large, small, or islets with different sizes, containing from 10 to 60 islets in a batch, were loaded into a flow chamber and continuously perifused for another 30 min with the same buffer at flow rate of 1 ml/min at 37°C, and then stimulated with KRH buffer containing 20 mM glucose. Perifusion fractions were collected every 3 min for 42 min, starting at 6 min prior to switching to 20 mM glucose. Insulin concentration in fractions was measured using Mouse Insulin ELISA (Mercodia). After each perifusion experiment, the chamber with islets was examined under Ti-E Inverted Nikon Microscope, and the acquired images were used for subsequent morphometric assessment of islets. The volume of isolated islets was evaluated from the measurement of two diameters and assuming that they have an ellipsoidal shape [26].

### Statistical analysis

The data are presented as means ± SEM. Comparisons of data were made by using two-tailed Student's t-test for independent data. The level of statistical significance was set at p<0.05.

## Results

### Abnormal development of pancreatic islets in p-KO mice

**Figure 1:**
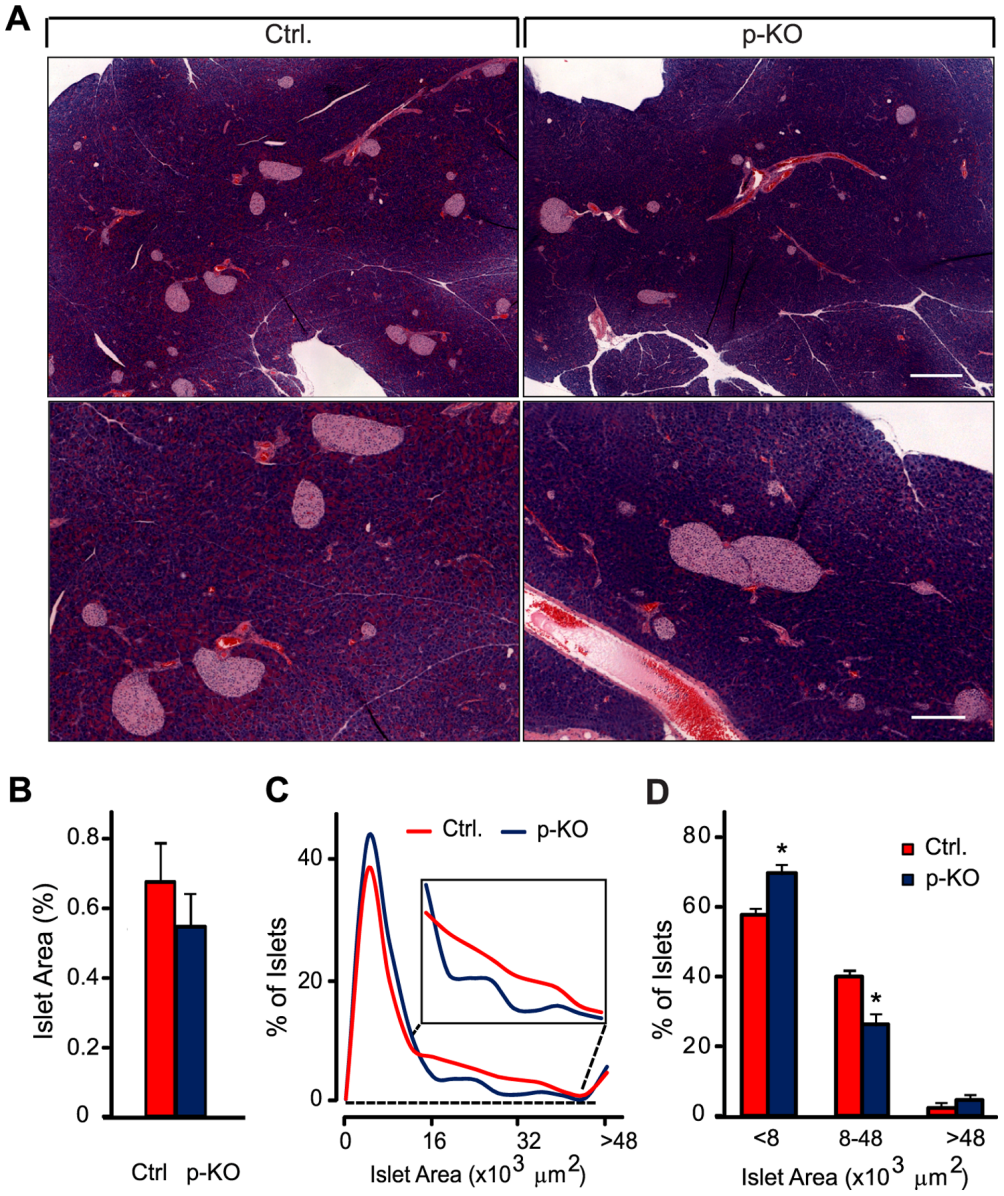
Pancreas-specific STAT3-KO (p-KO) mice exhibit altered pancreatic islet size distribution. **A.** Hematoxylin and eosin (H&E)-stained histological sections from 8-week-old control and p-KO mice. Composite images were obtained from multiple overlapping single images of histological sections. Scale bar  = 400 μm for upper and 200 μm for lower panels. **B.** Islet area relative to the whole pancreatic area in p-KO (blue) and control (red) mice (N = 4 mice). Data are presented as means ± SEM. Ten composite images of each pancreas were used for analysis, four mice per group. **C.** Histogram showing islet distributions in p-KO (blue) and control (red) mice according to islet areas. **D.** Proportion of small, medium, and large islets in p-KO (blue bar) and control (red bar) mice. Data in C and D were obtained from 500–570 islets from pancreatic histological sections from four mice per genotype. The proportion of medium-sized islets was significantly lower in p-KO mice as compared with those in controls, while the number of small islets was increased. *p<0.05.

To investigate the role of STAT3 signaling in the development and function of pancreatic islets, we generated mice with pancreas-specific STAT3 deficiency by crossing STAT3^fl/fl^ mice with and without Pdx-Cre transgene. As previously reported [15], STAT3 was expressed in the pancreas of control mice at an abundant level but was reduced to barely detectable level in p-KO mice. In our previous study, female p-KO mice exhibited substantially impaired glucose tolerance, while male p-KO mice did not show the similar glucose intolerance, and responses from transgenic male mice were highly variable; therefore, female p-KO mice were previously used [15]. Here we extended our analysis and carried out further studies using p-KO and littermate STAT3^fl/fl^ (Ctrl.) female mice. To determine whether STAT3 deficiency affected pancreatic islet morphology, we performed histological analysis of pancreata specimens from p-KO and control mice. Representative images of pancreatic sections are shown in Fig. 1A. First, we evaluated the islet area relative to the whole pancreatic area, which is considered an important indicator of islet function [27], [28], in p-KO and control mice. The results of histological analysis showed that the relative islet area in p-KO and control mice were not significantly different (0.53±0.09% and 0.68±0.10%, respectively, N = 4 mice for each genotype, NS, Fig. 1B). Histological examination of pancreata specimens did not reveal any noticeable pathological changes in the pancreatic tissue of transgenic animals. Pancreatic sections from transgenic mice appeared to have fewer medium-sized islets, more islets of small size, and some very large islets as compared with the islets from control mice. To perform quantitative assessment of islet size distribution, we analyzed the results of islet area measurements carried out on 500–570 islets from four control and four p-KO mice. Pancreatic islets from all examined sections were assigned to one of three groups, according to the size of islet area: small islets with an area <8,000 μm^2^, medium-sized islets with an area 8,000–48,000 μm^2^, and large islets with an area >48,000 μm^2^. Consistent with our initial observations, p-KO mice had significantly fewer medium-sized islets but more islets of small size (Fig. 1C, 1D). Although p-KO mice also appeared to have a higher proportion of large islets, the difference was not statistically significant.

### Altered architecture of pancreatic islets in p-KO mice

**Figure 2:**
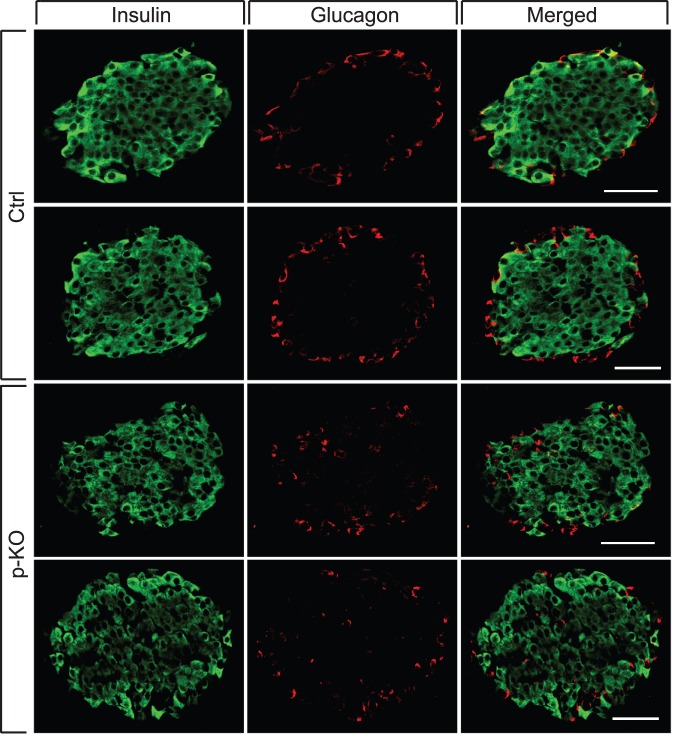
Pancreas-specific STAT3-KO (p-KO) mice exhibit altered pancreatic islet architecture. Immunohistochemical analyses of insulin (green) and glucagon (red) expression were performed on 5-μm pancreatic cryosections of p-KO and control mice. Insulin-positive β-cells (green) were located in the core of islets in both mouse lines. Glucagon-positive α-cells (red) were evenly distributed along the periphery of islets in control mice (Ctrl.); in p-KO mice the pattern of peripheral distribution of α-cells was disrupted, and some α-cells were scattered throughout islet core (p-KO). Scale bar  = 50 µm.

To investigate the effect of STAT3 deficiency on pancreatic islet architecture, we performed immunohistochemistry on pancreatic cryosections of control and p-KO mice. To detect the location of the most abundant β- and α-cells within islets, pancreatic sections were immunolabelled for insulin and glucagon (Fig. 2). In both mouse lines, insulin-positive β-cells were located in the central part of islets. Glucagon-positive α-cells were evenly distributed along the periphery of islets in control mice. However, in transgenic animals, this pattern of peripheral distribution of α-cells was disrupted, and some α-cells were scattered throughout islet core.

**Figure 3:**
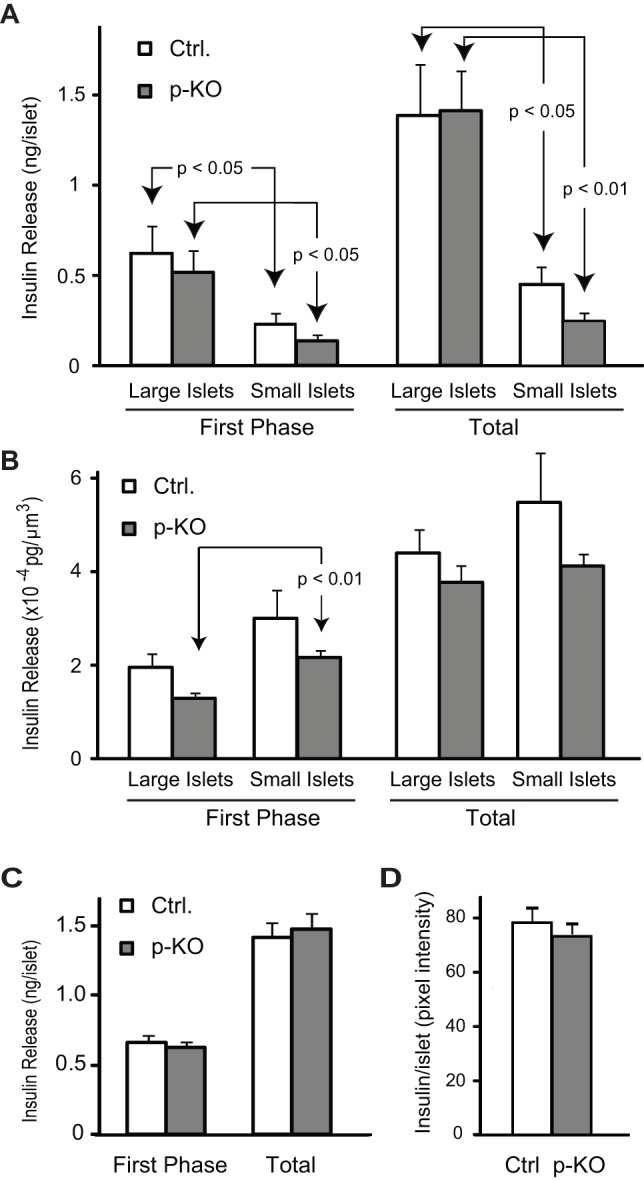
p-KO mice exhibit normal insulin secretion per unit volume. **A.** Groups of large and small isolated islets were separately examined for glucose-stimulated insulin secretion. Refer to the Results section for details on islet grouping. There was no difference in insulin secretion between p-KO and control islets of different sizes for the first phase or the entire stimulation period. Small islets secreted less insulin than large islets isolated from mice of the same genotype. **B.** Insulin secretion per unit volume. When insulin secretion data from **A** were normalized to islet size, there was no significant difference in insulin secretion between p-KO and control small islets as well as p-KO and control large islets during the first phase or the entire period of glucose-stimulated insulin release. Small p-KO islets secreted more insulin per unit of islet volume than large p-KO islets during the first phase (p<0.01) but not during the entire period of glucose-stimulated insulin release. **C.** Glucose-stimulated insulin secretion was measured in isolated islets from p-KO and control mice. Groups of 50-60 islets were incubated in KRH buffer containing 3 mM glucose (basal) before switching to 20 mM glucose (stimulatory). Net glucose-stimulated insulin secretion, were calculated as the sum of the insulin amount per islet in all fractions during the first 15 min (first phase) or the entire stimulation period (Total) after baseline subtraction. No difference was observed in the first phase or total insulin secretion between p-KO (grey bars, N = 3) and control (white bars, N = 3) mice. Data are presented as means ± SEM. D. Insulin content in pancreatic islets was assessed as the average pixel intensity of insulin positive signal per islet. Digital images were acquired with identical settings from 8–12 randomly selected pancreatic cryosections immunolabeled for insulin of each mouse. There was no difference in the intensity of insulin positive staining per islet in pancreatic sections obtained from control (white bar, N = 3) and p-KO mice (grey bar, N = 3).

To evaluate insulin content in islets, we labeled insulin in pancreatic cryosections and measured the intensity of insulin-positive staining in islets after background subtraction. As shown in Fig. 3D, there was no difference in islet insulin content between the genotypes.

### P-KO mice exhibit normal glucose-stimulated insulin secretion in isolated islets of different sizes *in vitro*

The finding that STAT3 deletion affected pancreatic islet development prompted us to examine insulin secretion from isolated islets in greater detail. To investigate whether the abnormal islet size distribution in p-KO mice along with reduced microvascular network could account for the delayed insulin response *in vivo* [15], we tested glucose-stimulated insulin secretion in batches of islets belonging to different size categories isolated from control and transgenic mice. For evaluation of glucose-induced insulin secretion *in vitro*, we calculated the amount of insulin secreted per islet in all fractions, collected during the first 15 min, corresponding to the first phase of insulin secretion, and during the entire period of stimulation (total) after subtraction of basal secretion. First, we assessed glucose-stimulated insulin secretion of small and large islets separately. We grouped isolated islets into large (3.4±0.9×10^6^ μm^3^ and 4.5±1.1×10^6^ μm^3^ for control and p-KO, respectively; N = 4–5 mice for each genotype) and small (0.9±0.1×10^6^ μm^3^ and 0.7±0.1×10^6^ μm^3^ for control and p-KO, respectively; N = 4–5 mice for each genotype) islets and tested their insulin secretion in response to 20 mM glucose. Both large and small islets secreted insulin efficiently. As expected, small islets secreted less insulin than large islets of the same genotype (Fig. 3A). No difference in insulin secretion was found between p-KO and control large islets during the first phase or the entire period of glucose-stimulated insulin release. Small islets from transgenic mice secreted marginally less insulin during the first phase of insulin secretion (p = 0.082) and the entire period of stimulation (p = 0.081) than islets from control littermates. To take into account the differences in islet sizes between groups of control and transgenic mice, we determined the amount of insulin secretion per unit of islet volume. When insulin secretion was normalized to islet volume, a similar trend was noted, however, there were no significant differences in insulin secretion between p-KO and control small islets as well as p-KO and control large islets during the first phase or the entire period of glucose-stimulated insulin release (Fig. 3B). Small islets from p-KO mice secreted more insulin per islet volume than large p-KO islets during the first phase (p<0.01, Fig. 3B) but not during the entire period of glucose-stimulated insulin release. To assess *in vitro* glucose-stimulated insulin secretion from islets representative of the diversity of islet size differences in p-KO and control mice, we measured insulin secretion in batches containing large number of randomly selected islets (>50 islets per mouse, N = 3 mice for each genotype). Similar to our previous perifusion studies performed on islets of similar size [15], we found no apparent difference in the first phase or the entire stimulation period between p-KO and control mice (Fig. 3C). Thus, the disruption of STAT3 signaling in the pancreas results in alterations in islet morphology without significant influence on glucose-stimulated insulin secretory function of isolated islets *in vitro*.

## Discussion

Our previous work shows that STAT3 signaling in the pancreas is essential for the development of pancreatic vasculature via its regulation of VEGF-A and that glucose intolerance and impaired insulin secretion in mice with pancreas-specific STAT3 deficiency are associated with microvascular alterations in the pancreas [15]. These mice exhibit glucose intolerance and impaired insulin secretion *in vivo*, but normal insulin secretory function in response to glucose challenge in isolated islets. In the present study, we extend our analysis and evaluate the role of STAT3 signaling in the development and maintenance of pancreatic islets.

Microscopic examination of histological sections revealed abnormal size distribution of pancreatic islets in p-KO mice: a higher proportion of small islets but a lower proportion of medium-sized islets in transgenic mice as compared with those in control pancreata. Due to a substantial lobular heterogeneity within the pancreas in terms of islet size distribution and functional properties [29], [30], [31], the analysis was performed for the dorsal pancreas of transgenic and control mice. Our findings are consistent with those in the previous VEGF-A–KO study by Lammert *et al*. [16]. In pancreas-specific, but not in β-cell-specific VEGF-A–KO mice, abnormal islet development, including increased number of small islets was reported; however, the total endocrine islet area relative to the whole pancreas remained similar to that in the control mice, indicating that VEGF-A from non-β-cells can compensate for the loss of VEGF-A from β-cells during islet development [16], [19]. Immunohistochemical analysis of pancreatic sections revealed normal peripheral distribution of α-cells [32] in control mice and abnormal pattern of α-cells location within islets of p-KO mice. Similar abnormalities in islet architecture in β-cell–specific STAT3-deficient mice, such as disturbed localization pattern of α-cells, were also described by Gorogawa and coauthors [33]. Hormone secretion in the endocrine pancreas is influenced by a number of circulatory factors, including islet hormones. For example, insulin inhibits glucagon secretion and possibly the secretion of other hormones, while glucagon stimulates the secretion of insulin and somatostatin [34], [35]. As hormones secreted by one cell type may have a regulatory effect on the secretory activity of another cell type, the order of endocrine cell perfusion in living islets is critical in determining the interaction of different types of endocrine cells in the regulation of blood glucose levels [5], [28], [36]. Two major islet blood flow patterns in the mouse pancreas were described by Nyman and co-authors [37]: the predominant inner-to-outer type of perfusion, where the core of beta-cells is upstream of the non beta-cell mantle, and the products of beta cell secretion likely regulate the secretory activity of non-beta-cells; the top-to-bottom blood flow pattern, found in 35% of islets, where the direction of blood flow is not dependant on cell types. We speculate that abnormal islet architecture in p-KO mice may result in altered order of endocrine cell perfusion and therefore has a regulatory effect on the secretory activity of other islet cells depending on blood flow patterns [28], [36] in pancreatic islets. However, future detailed studies are needed to address these specific and complex questions. These results suggest that islet development and maintenance depends on intact STAT3 and VEGF-A signaling pathways.

To evaluate a possible role in p-KO mice for the abnormal islet size distribution in reducing the *in vivo* insulin response and in glucose intolerance, we measured insulin secretion in batches of isolated islets of different sizes (>50 islets per mouse) in perifusion experiments. In our previous studies [15], we examined glucose-stimulated insulin secretion in groups of ∼20 similar-sized islets and found no apparent difference in the first phase or the entire stimulation period between p-KO and control mice. However, in our earlier experiments we used a relatively small number of medium-sized islets, and the choice of islets did not represent the different islet size distributions. To test whether altered islet size along with reduced pancreatic vascular network account for the delayed insulin response in p-KO mice *in vivo*, we performed *in vitro* perifusion experiments using batches of islets representative of isolated islet size distribution from control and p-KO mice. Consistent with our previous findings, these experiments did not reveal a noticeable difference in insulin secretion in the first phase or during the entire stimulation period between p-KO and control mice. To further examine this possibility, we compared glucose-stimulated insulin secretion from batches of small and large isolated islets separately. As expected, large islets secreted more insulin than smaller islets (∼15%–30% of the size of larger islets); however, when insulin secretion was normalized to unit volume of islets, large and small islets secreted comparable amounts of insulin. Similar findings were reported by Colella at al. in experiments with isolated rat islets of different sizes [38]. It was shown that larger islets utilized more glucose than smaller islets; however, the amount of insulin secreted per unit DNA content in response to glucose was the same in islets of all sizes. Considering that the islet percentage relative to total pancreas was not different between p-KO and control mice, it is conceivable that the amount of insulin secretion would not be different between the two genotypes if all the islets were isolated and measured *in vitro*. Together with previous molecular and cellular analyses on glucose uptake, glucose metabolism, and calcium responses in pancreatic islets, these data establish that the insulin secretory process is normal in p-KO mice and that impaired insulin response *in vivo* and glucose intolerance in the absence of insulin resistance are unlikely to be caused by intrinsic β-cell secretory defects or altered islet distribution. Thus, the different islet size distribution found in p-KO mice could not account for the delayed insulin response *in vivo.*

Recent studies showed that Pdx1-Cre transgenic lines exhibit restricted expression of Cre recombinase in specific regions of the brain including the hypothalamus [39], [40], thus raising the possibility that STAT3 may be inadvertently deleted in the p-KO mouse hypothalamus. Given the importance of hypothalamic STAT3 signaling in the regulation of energy homeostasis [41], potential loss of STAT3 signaling in hypothalamic neurons may contribute to the phenotype exhibited by p-KO mice. However, the relative contribution of local microvascular alterations in the pancreas and systemic changes associated with altered STAT3 expression in the hypothalamus should be further investigated. In summary, we studied pancreatic STAT3 function by generating a conditional STAT3 deletion with Pdx1-driven Cre expression, and demonstrated that STAT3 signaling in the pancreas is required for normal islet development and growth. Altered islet architecture and abnormal pattern of islet size distribution in transgenic mice do not affect islet secretory function *in vitro* and, therefore, does not play a significant role in glucose intolerance and impaired insulin secretion exhibited by p-KO mice *in vivo*. Our findings support a functional role for pancreatic STAT3 signaling in the normal development and maintenance of endocrine pancreas.
